# Supplementary material for: Effect of formulations over a Periodic Capacitated Vehicle Routing Problem with multiple depots, heterogeneous fleet, and hard time-windows
Source: PLoS One. 2024 Oct 3;19(10):e0311303. doi: 10.1371/journal.pone.0311303 (PMC11449318; doi:10.1371/journal.pone.0311303)
Supplement: S1 Appendix — (PDF) [file pone.0311303.s001.pdf]

Appendix.

Constraints (46) guarantee the consistency of variables x_{ij}^{h} and w_i^{kh}. This means that if one of w_i^{kh} and w_j^{kh} variables is one and the other is zero, x_{ij}^{h} must be zero as it is not feasible to go from node i to node j on day h as the same vehicle do not serve both clients.

If $w_i^{kh} = 1$ and $w_j^{kh} = 0$, constraint (46) is reduced to $x_{ij}^{h} \leq 0$. The problem arises when $w_i^{kh} = 0$ and $w_j^{kh} = 1$ because constraint (46) is reduced to $x_{ij}^{h} \leq 2$. The purpose of this demonstration is to show that, if $w_i^{kh} = 0$ and $w_j^{kh} = 1$, x_{ij}^{h} must be zero.

We will proof the statemen using *reductio ad absurdum*. We will drop supraindex h as all the involved constraints are for all possible days so it is redundant. Let us suppose that there are n different vehicles. We can define set $K = \{k_1, k_2, ..., k_n\}$. Let us fix the serving vehicle as k_1. From the statement we have that $w_i^{k_1} = 0$ and $w_j^{k_1} = 1$. Let us suppose that $x_{ij} = 1$.

As $x_{ij} = 1$, according to constraints (42), $\sum\limits_{k \in K} w_j^k = 1$ because client j is being visited. But $w_j^{k_1} = 1$, then $\sum\limits_{\substack{k \in K \\ k \neq k_1}} w_j^k = 0$. This means that $w_j^{k_2} = w_j^{k_3} = w_j^{k_n} = 0$. In the same line of thought, if $x_{ij} = 1$, then client i was visited prior to client j, then $\sum\limits_{k \in K} w_i^k = 1$. But $w_i^{k_1} = 0$, then $\sum\limits_{\substack{k \in K \\ k \neq k_1}} w_i^k = 1$. Let us assume that the vehicle serving client i is k_2, then $w_i^{k_2} = 1$.

Let us analyze constraints (46) for vehicle k_2: $x_{ij} \leq 1 - w_i^{k_2} + w_j^{k_2}$. We already established that $w_i^{k_2} = 1$ and $w_j^{k_2} = 0$. Then the inequality turns into $x_{ij} \leq 0$ which implies that $x_{ij} = 0$. But this is a contradiction because it was assumed that $x_{ij} = 1$.

We can conclude that if $w_i^{kh} = 0$ and $w_j^{kh} = 1$, x_{ij}^{h} must be zero.
