# Supplementary material for: Effect of formulations over a Periodic Capacitated Vehicle Routing Problem with multiple depots, heterogeneous fleet, and hard time-windows
Source: PLoS One. 2024 Oct 3;19(10):e0311303. doi: 10.1371/journal.pone.0311303 (PMC11449318; doi:10.1371/journal.pone.0311303)
Supplement: Attachment — Submitted filename: Response to reviewers.pdf [file pone.0311303.s002.pdf]

Medellín, 5th July of 2024

Academic Editor: Yangming Zhou, Ph.D.
PLOS ONE

Dear Academic Editor,

First of all, we want to thank you, and the reviewers for all the valuable comments received about the manuscript of the paper "Effect of formulations over a Periodic Capacitated Vehicle Routing Problem with Multiple Depots, Heterogeneous Fleet, and Hard Time-Windows." We found them very useful and we changed the structure of the paper to comply with the observations.

We have prepared an improved paper version, considering the reviewers' comments, which we will explicitly reply to next. All the changes are written in blue. The section "Performance comparison between formulations" was completely changed.

General comments:

Comment (C)/ "The paper is well-written. The authors should clarify how they produce the instances and ensure their accessibility. In addition, additional experimental evaluation is necessary to reinforce the main contribution. All reviewers'comments should be carefully considered and solved in your revised manuscript."

Response (R)/ Section "Performance comparison between formulations" now clarifies how the instances were produced. Also, a more complete experimentation was done and analyzed. In addition, a real-life case study data set was added.

C/ "Journal requirements."

R/ All the journal requirements are now met (PLOS ONE's style, code sharing guidelines, funding is not in acknowledgments anymore, instances are available).

C/ 1. Is the manuscript technically sound, and do the data support the conclusions?

The manuscript must describe a technically sound piece of scientific research with data that supports the conclusions. Experiments must have been conducted rigorously, with appropriate controls, replication, and sample sizes. The conclusions must be drawn appropriately based on the data presented.

Reviewer #1: No

Reviewer #2: Yes

R/ A more complete experimentation was performed and included in section "Performance comparison between formulations."

C/ 2. Has the statistical analysis been performed appropriately and rigorously?

Reviewer #1: No

Reviewer #2: Yes

R/ In the section "Performance comparison between formulations," the different possible factors that might influence the results were taken into account.

C/ 3. Have the authors made all data underlying the findings in their manuscript fully available?

The PLOS Data policy requires authors to make all data underlying the findings described in their manuscript fully available without restriction, with rare exception (please refer to the Data Availability Statement in the manuscript PDF file). The data should be provided as part of the manuscript or its supporting information, or deposited to a public repository. For example, in addition to summary statistics, the data points behind means, medians and variance measures should be available. If there are restrictions on publicly sharing data—e.g. participant privacy or use of data from a third party—those must be specified.

Reviewer #1: Yes

Reviewer #2: Yes

R/ Additional to tables in the paper, all the instances were made available on GitHub.

C/ 4. Is the manuscript presented in an intelligible fashion and written in standard English?

PLOS ONE does not copyedit accepted manuscripts, so the language in submitted articles must be clear, correct, and unambiguous. Any typographical or grammatical errors should be corrected at revision, so please note any specific errors here.

Reviewer #1: Yes

Reviewer #2: Yes

R/ All the changes made to the original manuscript followed the same writing style as the original manuscript.

C/ Reviewer #1: The authors introduced two different but equivalent formulations for a PVRP with time windows, multiple depots, and heterogeneous fleet, considering all the

particularities associated with a real-life scenario in the vending machine industry in Medellin, Colombia. The authors also performed some experiments to comprehend if the two equivalent formulations bear different performances in GUROBI.

1.The studied topic is practical. The paper is well-written. But the main contribution of this work is to propose two formulations based on classical VRP models for a real-life problem. The workload of the paper seems not enough. I suggest this work to be published in another journal.

R/ Additional to the new formulations of the model, the proposed problem is new and conclusions over the performance of both formulations are presented.

We also added a more complete experimentation to further strengthen our conclusions. We also presented the real-life case study data set.

C/ Reviewer #2: The authors present a novel contribution to the Periodic Vehicle Routing Problem (PVRP) by introducing two new problem formulations that differ in the usage of the crucial flow variable. Experiments have demonstrated their impact on the performance of the GUROBI solver. The topic is interesting, and the manuscript is well-structured. There is only a small issue: the authors should explain in detail how they produce the instance data and ensure its accessibility. Additionally, more information is needed regarding the results on the real cases mentioned in the paper.

R/ The process to generate the instances is now detailed and accessible via GitHub. Additionally, information about the real case instances is provided in the second subsection of "Performance comparison between formulations."

C/ While revising your submission, please upload your figure files to the Preflight Analysis and Conversion Engine (PACE) digital diagnostic tool, https://pacev2.apexcovantage.com/. PACE helps ensure that figures meet PLOS requirements. To use PACE, you must first register as a user. Registration is free. Then, login and navigate to the UPLOAD tab, where you will find detailed instructions on how to use the tool. If you encounter any issues or have any questions when using PACE, please email PLOS at figures@plos.org. Please note that Supporting Information files do not need this step.

R/ Images not generated using latex (Fig 1 and 3) were uploaded after being adapted in PACE.

Best Regards,

Articles's authors.
